# Protein engineering for feedback resistance in 3-deoxy-D-*arabino*-heptulosonate 7-phosphate synthase

**DOI:** 10.1007/s00253-022-12166-9

**Published:** 2022-09-16

**Authors:** Kumaresan Jayaraman, Natalia Trachtmann, Georg A. Sprenger, Holger Gohlke

**Affiliations:** 1 Institut für Pharmazeutische und Medizinische Chemie, Heinrich-Heine-Universität Düsseldorf, 40225 Düsseldorf, Germany; 2 Institute of Microbiology, University of Stuttgart, Allmandring 31, 70569 Stuttgart, Germany; 3 Jülich Supercomputing Centre (JSC), Institute of Biological Information Processing (IBI-7: Structural Biochemistry), John von Neumann Institute for Computing (NIC), & Institute of Bio- and Geosciences (IBG-4: Bioinformatics), Forschungszentrum Jülich GmbH, 52425 Jülich, Germany

**Keywords:** Shikimate pathway, DAHP synthase, Enzyme engineering, Feedback resistance, Protein stability

## Abstract

**Abstract:**

The shikimate pathway delivers aromatic amino acids (AAAs) in prokaryotes, fungi, and plants and is highly utilized in the industrial synthesis of bioactive compounds. Carbon flow into this pathway is controlled by the initial enzyme 3-deoxy-D-*arabino*-heptulosonate 7-phosphate synthase (DAHPS). AAAs produced further downstream, phenylalanine (Phe), tyrosine (Tyr), and tryptophan (Trp), regulate DAHPS by feedback inhibition. *Corynebacterium glutamicum*, the industrial workhorse for amino acid production, has two isoenzymes of DAHPS, AroF (Tyr sensitive) and AroG (Phe and Tyr sensitive). Here, we introduce feedback resistance against Tyr in the class I DAHPS AroF (AroF_cg_)*.* We pursued a consensus approach by drawing on structural modeling, sequence and structural comparisons, knowledge of feedback-resistant variants in *E. coli* homologs, and computed folding free energy changes. Two types of variants were predicted: Those where substitutions putatively either destabilize the inhibitor binding site or directly interfere with inhibitor binding. The recombinant variants were purified and assessed in enzyme activity assays in the presence or absence of Tyr. Of eight AroF_cg_ variants, two yielded > 80% (E154N) and > 50% (P155L) residual activity at 5 mM Tyr and showed > 50% specific activity of the wt AroF_cg_ in the absence of Tyr. Evaluation of two and four further variants at positions 154 and 155 yielded E154S, completely resistant to 5 mM Tyr, and P155I, which behaves similarly to P155L. Hence, feedback-resistant variants were found that are unlikely to evolve by point mutations from the parental gene and, thus, would be missed by classical strain engineering.

**Key points:**

*• We introduce feedback resistance against Tyr in the class I DAHPS AroF*

*• Variants at position 154 (155) yield > 80% (> 50%) residual activity at 5 mM Tyr*

*• The variants found are unlikely to evolve by point mutations from the parental gene*

**Supplementary Information:**

The online version contains supplementary material available at 10.1007/s00253-022-12166-9.

## Introduction

The shikimate pathway (or general aromatic biosynthesis pathway) is a seven-step metabolic pathway found in microorganisms, fungi, and plants, but is missing in multicellular animals (Herrmann and Weaver 1999; Sprenger 2007). The pathway starts with the condensation of the two precursor substrates, phosphoenolpyruvate (PEP) and erythrose-4-phosphate (E4P), and ends with chorismate. From there, various pathways diverge, such as those leading to precursors of aromatic vitamins (e.g., folate, ubiquinone, menaquinone, vitamin E). Importantly, chorismate is converted to the three aromatic amino acids (AAAs), L-phenylalanine (Phe), L-tyrosine (Tyr), and L-tryptophan (Trp), which are vital for the growth of all organisms. Over time, ways to develop microbial producer strains of the three AAA by either classical strain breeding (random mutagenesis, followed by screening or selection with antimetabolites) or genetic/metabolic engineering (e.g., improving precursor supply, cloning and expression of selected pathway genes, removal of regulation tiers such as repression, attenuation, or feedback inhibition) have led to high titer production strains that are used on an industrial scale. As well, many other shikimate pathway-derived bioactive compounds and polymers have been developed (Bongaerts et al. 2001; Ikeda 2006; Lee and Wendisch 2017; Martinez et al. 2015; Park et al. 2021; Rodriguez et al. 2014; Sprenger 2007). For example, apart from shikimate production (Syukur Purwanto et al. 2018), the pathway is exploited for the biotechnological production of desired plant products such as resveratrol, reticuline, opioids, and vanillin (Bongaerts et al. 2001; Lee and Wendisch 2017; Rodriguez et al. 2014). *Escherichia coli* (*E. coli*) and *Corynebacterium glutamicum* (*C. glutamicum*) are the two major “workhorse” microorganisms that are used for industrial productions of AAA and shikimate pathway-derived compounds (Bongaerts et al. 2001; Ikeda 2006; Lee and Wendisch 2017; Rodriguez et al. 2014; Sprenger 2007; Syukur Purwanto et al. 2018).

3-Deoxy-D-*arabino*-heptulosonate 7-phosphate synthase (DAHPS) (EC 2.5.1.54) is the first enzyme of the shikimate pathway and catalyzes the reaction of PEP and E4P to 3-Deoxy-D-*arabino*-heptulosonate 7-phosphate (DAHP) and inorganic phosphate (Herrmann and Weaver 1999; Srinivasan and Sprinson 1959). DAHPS controls the carbon flow into the shikimate pathway of bacteria (Ogino et al. 1982). This is accomplished primarily through feedback inhibition (allosteric regulation) exerted by the end product AAAs (Cho et al. 2011; Light and Anderson 2013; Ogino et al. 1982; Sprenger 2007), although transcriptional control is also found (Brown and Somerville 1971; Herrmann and Weaver 1999; Sprenger 2007). While this ensures the economics of the cell’s metabolism, this feedback inhibition hampers the biotechnological production of desired compounds. Hence, introducing feedback-inhibition resistance (FBR) in DAHPS has been a long-standing goal. In the past, this has been achieved in classical strain breeding by random mutagenesis, followed by screening and selection for useful compounds (Ikeda 2006; Sprenger 2007). Selection could be by the growth of mutated strains on minimal media in the presence of antimetabolites such as methylated or halogenated AAAs (Ikeda 2006; Sprenger 2007). These antimetabolites behave as effectors like the natural AAAs. They bind to the allosteric site in a DAHPS, thereby inhibiting its enzyme activity, and, in turn, lead to an auxotrophy for AAAs. Mutants that carry alterations in the allosteric site of DAHPS may no longer bind both the antimetabolites and the natural AAAs, thus leading to feedback inhibition resistance and prototrophy. In genetic engineering, knowledge of the DAHPS structure (best with bound effector in the active site) allows us to alter genes purposefully as to obtain feedback-resistant forms of DAHPS.

In *C. glutamicum*, DAHPS occurs in two forms, type I and type II. Type II DAHPS is highly utilized for the production of AAAs (Chen et al. 1993; Liu et al. 2008). It is activated by binding of Trp and chorismate mutase. By contrast, it is feedback inhibited by Phe and Tyr (Burschowsky et al. 2018). Type I DAHPS is sensitive toward even lower amounts of Tyr (Liu et al. 2008). In this work, type I DAHPS from *C. glutamicum* (hereafter termed AroF_cg_) is studied. AroF_cg_ has 45–55% sequence identity with the three isoforms of DAHPS from *E. coli*, AroF_ec_, AroG_ec_, and AroH_ec_, which are feedback inhibited by Tyr, Phe, and Trp, respectively (Shumilin et al. 1999; Umbarger 1978).

As to AroG_ec_, variants with single substitutions D146N or P150L are completely feedback-resistant to Phe inhibition, whereas variants with M147I or A202T are partially resistant (Kikuchi et al. 1997). Combining D146 and M147 with A202T to make double variants [M147I, A202T] and [D146N, A202T] of AroG_ec_ also led to feedback resistance toward Phe at 20 mM (Ding et al. 2014). A recent study exposed that Gln151 is also involved in Phe inhibition of AroG_ec_ (Yenyuvadee et al. 2021). The variants S180F, P150L, L175D, L179A, F209A, F209S, or V221A are also found to be feedback-resistant in Phe-sensitive AroG_ec_ (Ger et al. 1994; Hu et al. 2003; Jiang et al. 2000).

X-ray crystal structures of AroF_ec_ (bound inhibitor Tyr) and AroG_ec_ (bound substrate PEP and inhibitor Phe) shed light on the enzymes’ catalytic and inhibitor sites and mechanisms (Shumilin et al. 2003, 2004, 1999, 2002). The binding of Phe induces conformational changes in AroG_ec_ by modifying polar and non-polar interactions within the inhibitor and catalytic binding sites (Shumilin et al. 2002).

Here, we set out to exploit this knowledge for structure-based protein engineering to induce feedback resistance in AroF_cg_. Based on sequence and structural analysis, the residues of the inhibitor binding site are predicted in a structural model of AroF_cg._ To induce feedback resistance in AroF_cg_, substitutions at the inhibitor binding site are predicted, transferring knowledge from the homologous enzymes in *E. coli* and assessing the effects on the protein stability with folding free energy calculations using FoldX (Schymkowitz et al. 2005) and Rosetta (Kellogg et al. 2011). Eight variants were predicted and evaluated in vitro. The AroF_cg_ variants E154N and P155L are more than 80% and 50% feedback-resistant and active even in the presence of 5 mM Tyr.

## Materials and methods

### Homology modeling of AroF_cg_

AroF_cg_ (Uniprot ID: P35170) is sequentially similar to homologous enzymes expressed in *E. coli* and other organisms (Liu et al. 2008; Shumilin et al. 2004, 1999). No structure has been experimentally resolved for AroF_cg_. Here, we generated a comparative model of AroF_cg_ using the in-house program TopModel (Mulnaes et al. 2020) with default mode, which selected multiple template structures from *E. coli* and other organisms (Supplementary Table S1). The sequence identities, similarities, and coverages of the template sequences with respect to the target sequence are summarized in Supplementary Table S1. The model quality was assessed with TopScore (Mulnaes and Gohlke 2018) available from the TopSuite webserver (Mulnaes et al. 2021). The overall TopScore of the structural model is 0.1661, indicating a high quality of the model. The residue-wise TopScore shows that the core region is modeled with high quality (Supplementary Fig. S1). No template information was available for the N-terminal region (residues 1–25), however, such that no reliable structural model was generated for this region. For computations of folding free energies (see below), this region was not considered.

### Protein structure preparation

The generated AroF_cg_ model was prepared for stability predictions using the protein preparation wizard (Schrödinger Release 2018–1: Protein Preparation Wizard) of the Maestro graphical user interface of the Schrödinger suite (Release 2018–1: Maestro, Schrödinger, LLC, New York, NY, 2018). In this step, bond orders are corrected, and hydrogens are added to all residues. The protonation states of ionizable residues were set for pH 7.5 based on pK_a_ values predicted with PROPKA (Rostkowski et al. 2011). Furthermore, a restrained minimization was performed to correct strained bonds, angles, and clashes. The resulting structural model was used for the subsequent computations.

### Computations of folding free energy change

To screen for variants with high structural stability, the difference in the folding free energy between variant and AroF_cg_ wild type was computed using the two force field-based methods FoldX (Schymkowitz et al. 2005) and Rosetta (Kellogg et al. 2011).

1 $$\mathrm{\Delta \Delta }G={\Delta G}_{\mathrm{variant}}-{\Delta G}_{\mathrm{wildtype}}$$

Both methods have been shown to perform well in protein engineering studies (Buss et al. 2018; Nisthal et al. 2019).

### FoldX

FoldX (version 5) (Schymkowitz et al. 2005) uses an empirical force field to calculate the folding free energy from contributions by van der Waals interactions, hydrogen bonding, electrostatics, solvation effects, and entropy estimates. The BuildModel function of FoldX was used to calculate ΔΔ*G* (Eq. (1)). As input structure, the minimized AroF_cg_ wild-type structure was used. During variant generation, the neighboring residues of a specific variant are subject to conformational change. As each variant involves different neighboring residues, a corresponding wild type for each variant is produced. Per variant, 50 wild-type and variant structures were generated, over which the final ΔΔ*G* result was averaged. The uncertainty in the computations is given as the standard error of the mean (SEM), i.e., standard deviation/$\sqrt{50}$.

### Rosetta

The stability of the variants with respect to the wild type was predicted by the ΔΔ*G*_monomer module of the Rosetta suite (Kellogg et al. 2011) using the REF15 scoring function (Alford et al. 2017). Similar to FoldX, the energy function consists of a weighted sum of various energy terms. Two steps are performed by the ΔΔ*G*_monomer module: pre-minimization and ΔΔ*G* calculation. The pre-minimization was done with harmonic distance restraints adjusted such that the standard deviation of the distance is 0.5 Å and applied between all pairs of C_α_ atoms within 9 Å of each other to reduce steric clashes. The ΔΔ*G* values were predicted using the “high-resolution protocol”; this protocol enables backbone relaxation. Fifty models of variant and wild-type structures, respectively, were generated for each intended variant. The rotamers of all the residues were repacked, followed by three rounds of gradient-based energy minimization of all sidechain and backbone atoms. As above, the distance restraints were applied on C_α_ atoms to restrain the backbone mobility during the minimization process. Finally, ΔΔ*G* (Eq. (1)) was calculated as the difference between the top-scoring variant and the wild type. For each variant, ΔΔ*G* values are calculated ten times that way, and the final value is given as average ± SEM (i.e., standard deviation/$\sqrt{10}$).

### Cloning, expression, and purification of the DAHPS and its enzyme variants

The gene *aroF*_cg_ encoding *C. glutamicum* type I DAHP synthase was amplified from the chromosomal DNA of the *Corynebacterium glutamicum* ATCC 13,032 strain using primers 1 and 2 (Supplementary Table S1). The DNA amplificate was digested with *Nde*I and *Bam*HI restriction enzymes and cloned into the pET28a expression vector under the control of the T7 promoter (Table 1). By cloning the *aroF* gene, a sequence encoding an N-terminal 6xHis sequence of the protein was introduced. The recombinant plasmids, containing the respective gene variants (wild type or mutants), were transformed into the *E. coli* BL21(DE3) pLysS strain. The recombinant proteins were overexpressed in cells growing in LB medium at 30 ℃. Gene expression was induced by adding 0.5 mM of IPTG (final concentration). After 18 h of induction (30 ℃), cells were harvested by centrifugation at 5000 rpm, 4 ℃. *E. coli* cells were resuspended in binding buffer (50 mM Tris–HCl pH 7.2; 300 mM NaCl; 10 mM imidazole), sonicated, and centrifuged at 14,000 rpm for 30 min to separate the cell-free extract and precipitate. Cell-free extracts were loaded onto nickel-chelating columns (Qiagen) for purification. Washing was done in two steps: with buffer containing 50 mM Tris–HCl pH 7.2; 300 mM NaCl; 20 mM of imidazole (10 volumes); and then with 40 mM of imidazole (10 volumes). Elution was performed using the same buffer containing 250 mM of imidazole. The proteins were about 80–90% pure, as observed on SDS-PAGE.

**Table 1:** Plasmids and strains used in this work

Plasmid	Relevant markers	Reference or source
pET28a	pBR322 *ori* of replication; Km^R^, and all vectors below	Novagen, Germany
pET28a-aroF_cg_wt	*aroF* gene cloned in *Nde*I/*Bam*HI site, 6His-fusion at C-end	This work
pET28a-aroF-E154N		This work
pET28a-aroF-E154A		This work
pET28a-aroF-E154R		This work
pET28a-aroF-E154D		This work
pET28a-aroF-E154C		This work
pET28a-aroF-E154Q		This work
pET28a-aroF-E154G		This work
pET28a-aroF-E154H		This work
pET28a-aroF-E154I		This work
pET28a-aroF-E154L		This work
pET28a-aroF-E154K		This work
pET28a-aroF-E154M		This work
pET28a-aroF-E154F		This work
pET28a-aroF-E154P		This work
pET28a-aroF-E154S		This work
pET28a-aroF-E154T		This work
pET28a-aroF-E154W		This work
pET28a-aroF-E154Y		This work
pET28a-aroF-E154V		This work
pET28a-aroF-P155L		This work
pET28a-aroF-N156I		This work
pET28a-aroF-Q159A		This work
pET28a-aroF-T220V		This work
pET28a-aroF-D163A		This work
pET28a-aroF-S188F		This work
pET28a-aroF-D222A		This work
Strain	Genotype	
*E. coli* K-12 DH5α	F- Φ80d*lacZ*∆M15 ∆ (*lacZYA-argF*) U169 *rec*A1 *end*A1 *hsd*R17(rk-, mk +) *pho*A *sup*E44 λ - *thi*-1 *gyr*A96 *rel*A	Lab stock
*E. coli* B BL21(DE3) pLysS	F–, *omp*T, *hsd*S_B_ (r_B_–, m_B_–), *dcm*, *gal*, λ(DE3), pLysS, Cm^r^	Novagen, Germany

### Site-directed mutagenesis

Based on the protein modeling data, eight residues (E154, D163, S188, D222, P155, N156, Q159, T220) were selected. Site-directed mutagenesis was performed to study the contribution of these residues to the feedback resistance. The mutants E154N, D163A, S188F, D222A, P155L, N156I, Q159A, and T220V were prepared by the QuikChange PCR method using specific primer pairs (Supplementary Table S2). As a template, plasmid DNA containing the cloned *aro*F_cg_-wt gene (pET28a-aroF-wt) was used. The PCR was done using PfuUltra High-fidelity DNA Polymerase (Agilent, Germany). The residue E154 was replaced by the remaining 19 amino acid residues. All plasmids with mutated genes were transformed into BL21(DE3) pLysS, and proteins were overexpressed, purified, and activity assays were performed.

### Enzyme activity assays

The DAHP synthase activity was determined using two different assay methods, one continuous spectrophotometric (Jossek et al. 2001) and one discontinuous colorimetric method (Liao et al. 2001). The unit of DAHP synthase activity was defined as the disappearance of 1 µmole of phosphoenolpyruvate or the production of 1 µmole of DAHP per minute, respectively.

The colorimetric assay (Liao et al. 2001) was carried out in a final volume of 75 µl containing 5 mM PEP, 5 mM E4P, and Tris–HCl buffer (50 mM, pH 7.5). The reaction was initiated by the addition of 1–2 µg of the DAHP synthase preparation, incubated at 30 ℃ for 5 min, and stopped by the addition of 400 µl of 10% (w/v) trichloroacetic acid. The enzymatically produced DAHP was oxidized with NaIO_4_, and the product of this reaction (α-keto-butyrylaldehyde acid) was reacted with thiobarbituric acid at 100 ℃ to produce a pink chromophore. The absorbance of the chromophore was measured spectrophotometrically at 549 nm (ε = 45,000 M^−1^ cm^−1^) (Liao et al. 2001).

The continuous spectrophotometric assay was based on the disappearance of the phosphoenolpyruvate absorbance (λ = 232 nm; ε = 2800 M^−1^ cm^−1^). The reaction was done as described earlier in ref. (Jossek et al. 2001) (50 mM 1,3-bis[tris(hydroxymethyl)methylamino]propane (BTP) buffer, pH 6.8, 500 µM phosphoenolpyruvate, 500 µM E4P, 1 mM MnCl_2_). The reaction was initiated by adding 1–2 µg protein and carried out at 30 ℃. In feedback inhibition studies, the aromatic amino acid L-Tyr as an effector was added to the reaction mix.

### Sequence accession numbers

Sequences of the constructs used here have been deposited in NCBI Genbank (Supplementary Table S3).

## Results

### Structure-based prediction of feedback inhibition-resistant variants

To introduce resistance against feedback inhibition due to binding of Tyr to AroF_cg_, initially, variants were predicted by exploiting structural knowledge of homologous enzymes, sequence and functional data, a generated homology model of AroF_cg_, and folding free energy computations.

**Fig. 1:**
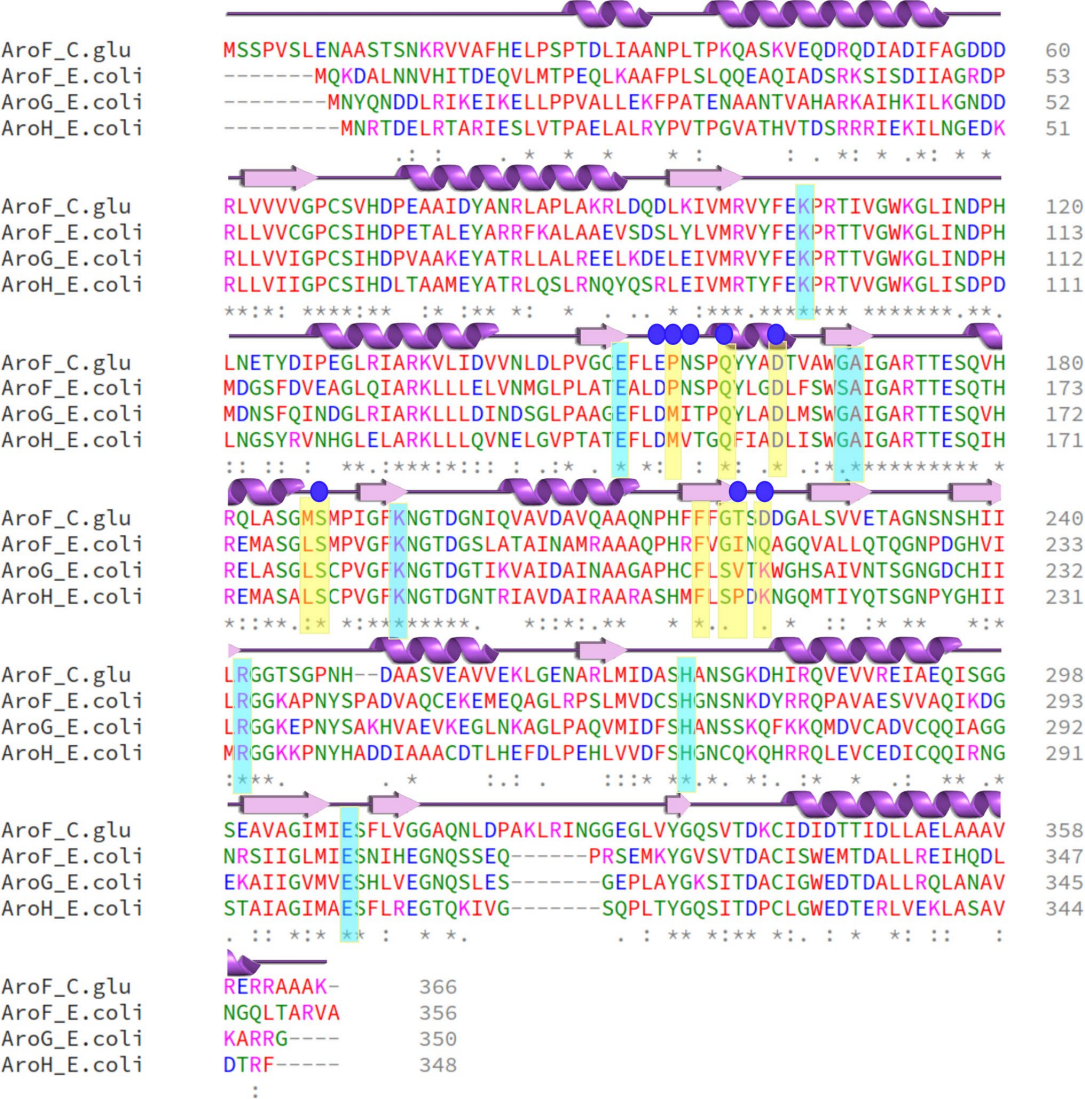
Multiple sequence alignment (MSA) of DAHPS. The MSA of AroF_cg_ with the isoforms of *E. coli* (AroF, AroG, and AroH) points out conserved regions (marked by “*”; “:” indicates similarity). The cyan and yellow boxes denote residues of the catalytic and feedback inhibitor binding sites. Residues subjected to ΔΔ*G* calculations are marked with filled blue circles above the sequence alignment. The secondary structure information of AroF_cg_ is provided on the top, obtained using PDBSUM (Laskowski 2001) on the modeled AroF_cg_

**Fig. 2:**
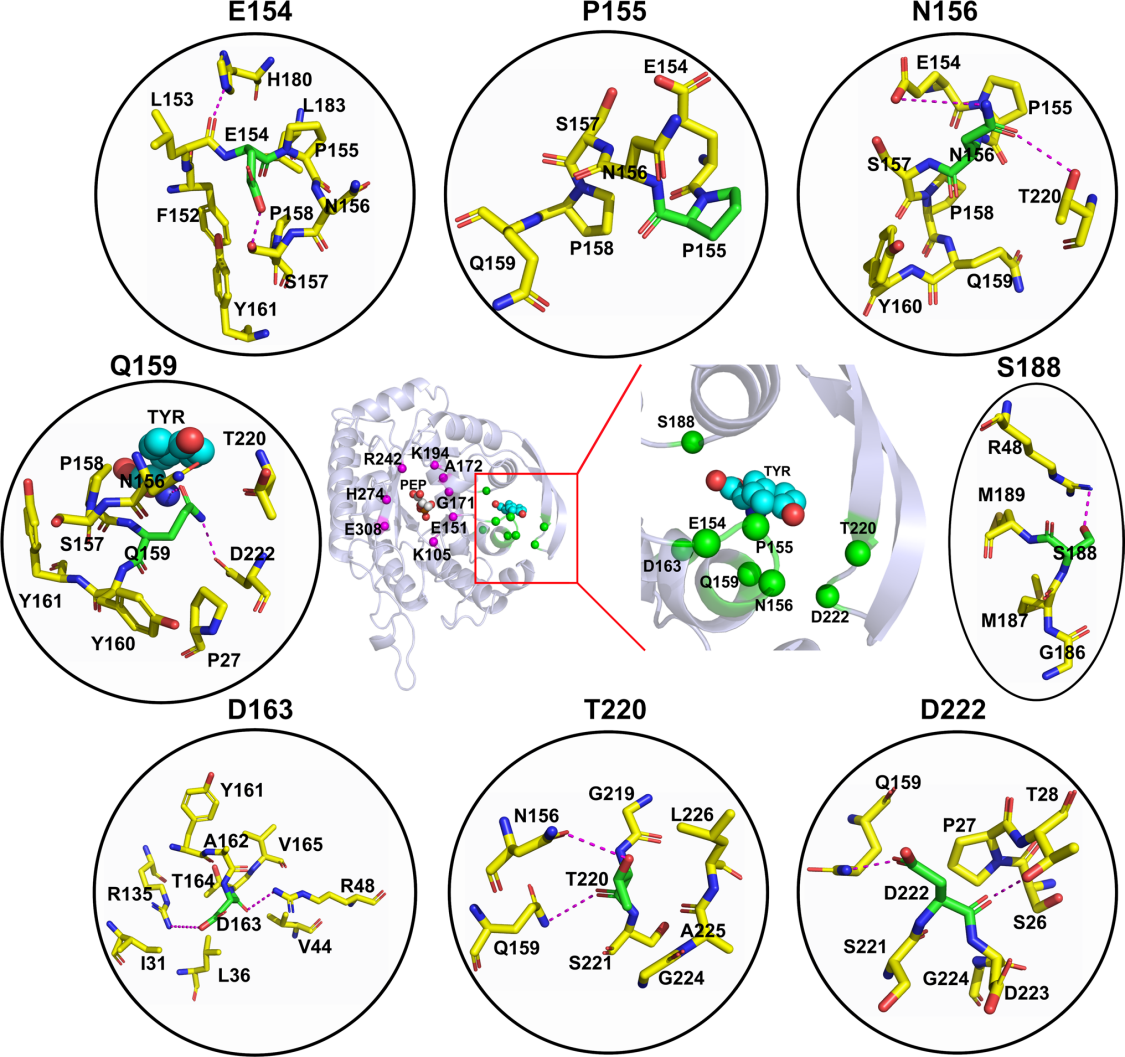
Residues of AroF_cg_ for which feedback-resistant variants were predicted. The structure of AroF_cg_ is depicted in cartoon representation in the middle. The C_α_ atoms of residues for which variants with feedback resistance were predicted and evaluated in ∆∆*G* computations are marked with green spheres. The C_α_ atoms of inferred catalytic site residues are marked with magenta spheres. The position of TYR is predicted by superimposing the crystal structure of Tyr-sensitive AroF_ec_ (PDB ID 6AGM). In the black circles, residues present around a variant position (green sticks) with ≤ 4 Å distance are shown (yellow sticks). Polar interactions between residues are denoted as magenta dashes

A multiple sequence alignment (MSA) was generated using Clustal Omega (Sievers et al. 2011) with the sequences of AroF_cg_ and those from three isoforms of *E. coli* DAHPS, AroF_ec_, AroG_ec_, and AroH_ec_. The sequence identity of AroF_cg_ with respect to the other three sequences is 47.3–52.5% (Supplementary Table S1). From the multiple sequence alignment and additional knowledge from the literature (Shumilin et al. 2004, 2002) on DAHPS enzymes, the catalytic site-forming residues are K105, E151, G171, A172, K194, R242, H274, and E308; residues of the inhibitor binding site are P155, Q159, D163, M187, S188, F217, G219, T220, and D222 (Fig. 1). Cocrystal structures of AroG_ec_ (PDB ID 1KFL) and AroF_ec_ (PDB ID 6AGM) with the feedback inhibitors Phe and Tyr, respectively, have been resolved (Supplementary Fig. S2 and S3) (Cui et al. 2019; Shumilin et al. 2002). A generated homology model of AroF_cg_ (Supplementary Fig. S1) was superimposed onto these cocrystal structures, which confirmed the residues forming the catalytic and inhibitor binding sites (Fig. 2). Most of the catalytic and inhibitor site residues are conserved. Both sites are at least 8.5 Å apart (Fig. 2). In the following, eight AroF_cg_ variants are predicted with putatively reduced feedback inhibition, which were chosen based on structural, sequence, and functional information.

**E154N:** This position is semi-conserved as the equivalent residues in *E. coli* DAHPS are aspartic acids. In AroG_ec_, the equivalent residue D146 interacts with T149 upon Phe binding (Shumilin et al. 2002) (Supplementary Fig. S2). In AroF_cg_, the interaction is between E154 and S157 (Fig. 2) and likely stabilizes the inhibitor binding region. Substitution of D146 with asparagine in AroG_ec_ resulted in complete resistance against feedback inhibition (Kikuchi et al. 1997). Hence, we chose E154N as an equivalent substitution for AroF_cg_.

**P155L:** P155 is predicted to interact with the inhibitor Tyr at the inhibitor binding site as equivalent residues in AroG_ec_ (M147) (Supplementary Fig. S2) and AroF_ec_ (P148) (Supplementary Fig. S3) interact with Phe and Tyr, respectively. An M147I variant in AroG_ec_ resulted in partial resistance to Phe inhibition (Kikuchi et al. 1997). In AroF_cg_, replacing P155 with a non-polar residue with a bulky side chain such as Leu will occupy more space in the inhibitor site, which likely hampers Tyr binding.

**N156I:** N156 of AroF_cg_ interacts with E154 and T220 to stabilize the binding pocket (Fig. 2). In AroH_ec_, the variant V147M at the equivalent position is feedback inhibition-resistant to Trp (Ray et al. 1988). Thus, replacing N156 with bulky aliphatic amino acids such as Ile should prevent the formation of polar contacts and likely destabilize the inhibitor site.

**Q159A:** Q159 is a conserved residue (Fig. 1) and is predicted to interact with the Tyr main chain at the inhibitor binding site (Fig. 2) because the equivalent residues in AroG_ec_ (Q151) (Supplementary Fig. S2) and AroF_ec_ (Q152) (Supplementary Fig. S3) interact with Phe and Tyr, respectively. As the nature of the interaction with Tyr is polar, replacing Q159 with a non-polar residue with a small side chain such as Ala will hinder the interaction, leading to feedback inhibition resistance.

**D163A:** D163 is a conserved residue and stabilizes the region by forming a salt bridge with R135 (Fig. 2). The equivalent residue in AroG_ec_ is D155 (Supplementary Fig. S2), which forms a salt bridge with K127. In AroF_ec_, it is D156 (Supplementary Fig. S3), which forms a salt bridge with K128. To abolish the salt bridge formation, D163 is substituted with Ala, which will destabilize this region and hamper Tyr binding.

**S188F:** S188 is conserved across the species. In AroG_ec_ and AroF_ec_, the Phe and Tyr main chains interact with the side chains of the equivalent residues S180 and S181, respectively (Supplementary Figs. S2 and S3). The S180F variant is resistant to feedback inhibition (Ger et al. 1994) because Phe abolishes the polar contacts and constricts the inhibitor site. Hence, the same substitution was adapted in AroF_cg_ to induce feedback inhibition resistance to Tyr.

**T220V:** T220 is part of the hydrophilic region of the inhibitor site and interacts with N156 and Q159 (Fig. 2), that way stabilizing the inhibitor site. Substituting T220 with Val will abolish these polar contacts and induce Tyr resistance.

**D222A:** D222 is proximal to Q159 and T28 (Fig. 2), and variants in this position with non-polar amino acids will destabilize the inhibitor site. In AroG_ec_, it corresponds to K214, which directly interacts with Phe (Supplementary Fig. S2). Hence, substituting D222 with Ala should hamper Tyr binding.

The predicted variants were assessed with FoldX and Rosetta with respect to changes in the total free energy compared to the wild-type AroF_cg_ (ΔΔ*G*, Eq. (1), Table 2). Variants were considered stable if at least one ΔΔ*G* value < 0 (Kellogg et al. 2011; Schymkowitz et al. 2005). This criterion is fulfilled by seven of the eight variants predicted above; S188F was nevertheless considered for experimental validation because of the strong indication from the literature (Ger et al. 1994).

**Table 2:** The changes in the folding free energy change (ΔΔ*G*) of predicted variants of AroF_cg_

Variants	ΔΔ*G*^[a]^	
	FoldX	Rosetta
*E154N*	0.83 ± 0.10	− 0.51 ± 0.77
*P155L*	− 0.50 ± 0.02	− 0.50 ± 0.90
*N156I*	− 0.23 ± 0.01	− 0.79 ± 0.88
*Q159A*	− 0.01 ± 0.04	− 1.96 ± 0.34
*D163A*	0.43 ± 0.02	− 0.88 ± 1.12
S188F	0.32 ± 0.00	0.49 ± 0.64
*T220V*	− 0.76 ± 0.01	− 2.58 ± 0.43
*D222A*	− 0.57 ± 0.04	− 3.29 ± 0.94

In kcal mol^−1^, ΔΔ*G* (Eq. 1) is predicted with FoldX and Rosetta. Given is the average ± SEM over *n* = 50 data points for FoldX and *n* = 10 for Rosetta. Variants in which at least one ∆∆*G* value < 0 are considered stable (marked in italics)

### In vitro studies on the predicted variants

We mutated the *aroF*_cg_ gene at the positions which had been proposed by the predictions (Table 2). Using the QuikChange methodology (for details, see the “Materials and methods” section), all gene variants were created and verified by DNA sequencing. The wild type and mutant proteins were provided with N-terminal 6xHis-fusions to allow fast purification by IMAC technology. The following variants from Table 2 could be successfully expressed (protein overexpression as visible in SDS-PAGE analysis) and purified by Ni–NTA affinity chromatography (Supplementary Fig. S4): E154N, P155L, N156I, Q159A, S188F, T220V, and D222A. Several attempts to express and purify the D163A variant, however, were unsuccessful (data not shown).

Next, we assayed the enzyme activity of DAHPS. One assay allows continuous measurement by a spectrophotometric method [40]. Another discontinuous measurement is by a colorimetric assay [41]. We noticed that the former assay allows to follow the kinetics of the reaction, but when the effector Tyr is added at concentrations above 500 µM, the method cannot be used as Tyr interferes with the photometric assay. To allow the addition of higher effector concentrations (e.g., 5 mM Tyr), we, therefore, switched to the discontinuous assay. Details are described in “Materials and methods.” Activity measurements with purified enzyme preparations gave similar values for the specific activity for both assay methods, although in some measurements, the absolute values were lower for the discontinuous assay (Supplementary Table S4). To allow comparisons of enzyme activities between the two methods, we, therefore, set the activity in the absence of the effector as 100% for each method. The feedback inhibitor Tyr was added to 50 µM (spectrophotometric assay) or 5 mM (colorimetric assay), and the remaining activity was set into relation to the 100% value.

### Feedback inhibition or resistance toward effector Tyr at 50 µM or 5 mM

**Fig. 3:**
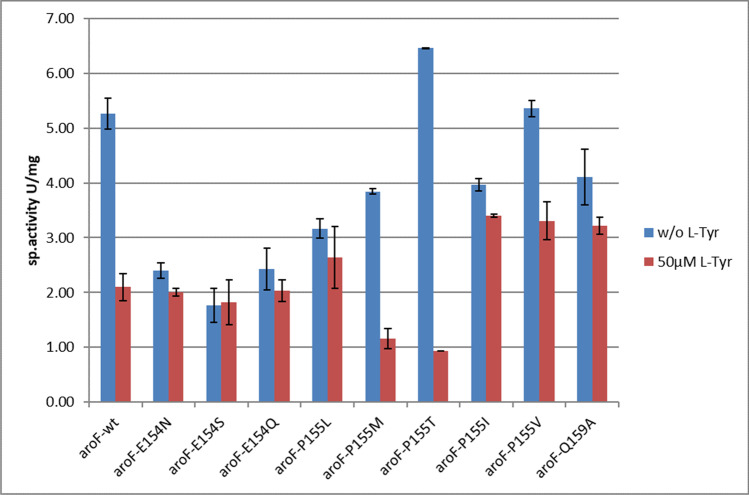
Comparison of wt AroF enzyme with variants at positions E154, P155, and Q159. Specific activity from spectrophotometric assays in the absence or presence of 50 µM Tyr as the effector

**Fig. 4:**
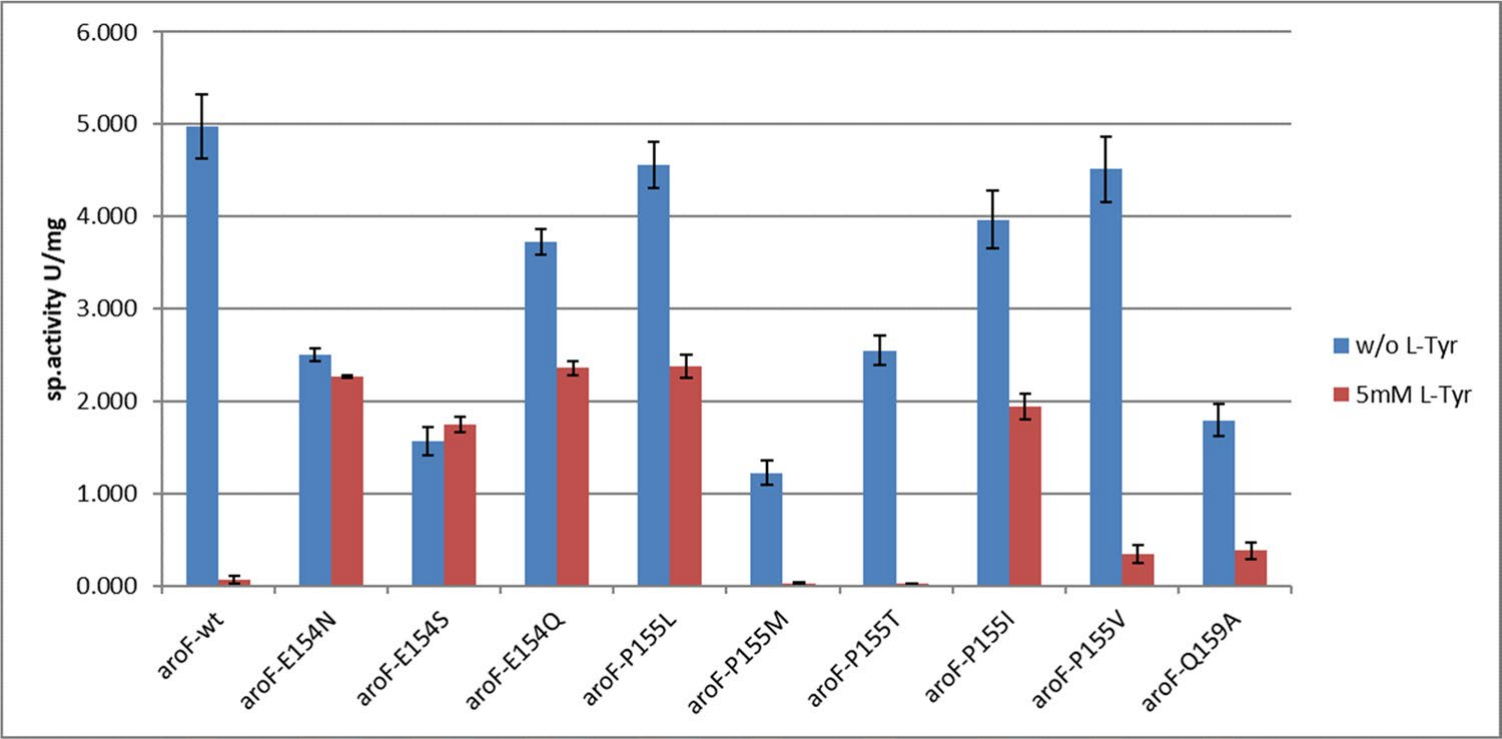
Comparison of DAHP synthase activities for wt AroF and variants at positions E154, P155, and Q159. Specific activity from the discontinuous, colorimetric assay in the absence or presence of 5 mM Tyr as the effector

The recombinant wild-type enzyme AroF_cg_ was very active under both assay conditions with specific activities of about 5 U/mg of protein (see Figs. 3 and 4 and Supplementary Table S4). With 50 µM of Tyr added, the residual activity was only about 40%, and at 5 mM Tyr, only negligible activity could be detected. We took this as proof that AroF_cg_ is feedback inhibited by Tyr already at physiological concentrations. For production processes, 5 mM Tyr would completely inhibit this enzyme. Variants N156I, T220V, and D222A displayed similar or lower activities than the wt enzyme, but were as sensitive to the addition of 50 µM of Tyr and were therefore not studied further (data not shown). Variant S188F showed less than 15% of wt activity in the absence of Tyr, but was not inhibited by 50 µM Tyr (data not shown).

Variants E154N, P155L, and Q159A were all less active than the wt enzyme in the absence of Tyr, but kept more than about 80% of their activities in the presence of 50 µM Tyr. Q159A lost activity when stored overnight at 4 ℃ and thus is very unstable. At 5 mM Tyr, it showed about 20% residual activity and was not considered for further measurements. E154N kept > 80% activity at 5 mM Tyr, P155L about 50% (Fig. 4).

We reasoned that the positions E154 and P155 were good candidates for an in-depth analysis. Therefore, further variants at these two positions were created: E154S, E154Q as well as P155M, P155T, P155I, and P155V. The prediction was that these amino acid changes, especially the hydrophobic residues at P155, could be candidates for feedback resistance toward Tyr. The folding free energy calculations of the variants predicted most of them as stable (Table 3).

**Table 3:** Changes of folding free energies of AroF_cg_ variants at positions 154 and 155

Variants	ΔΔ*G*^[a]^	
	FoldX	Rosetta
E154Q	1.13 ± 0.11	0.49 ± 0.37
*E154S*	1.99 ± 0.11	− 0.10 ± 0.65
*P155I*	0.55 ± 0.01	− 0.38 ± 0.87
*P155M*	− 0.81 ± 0.02	− 1.03 ± 0.78
P155T	1.41 ± 0.01	0.24 ± 0.73
*P155V*	1.55 ± 0.01	− 0.78 ± 0.62

In kcal mol^−1^, ΔΔ*G* (Eq. 1) is predicted with FoldX and Rosetta. Given is the average ± SEM over *n* = 50 data points for FoldX and *n* = 10 for Rosetta. Variants in which at least one ∆∆*G* value < 0 are considered stable (marked in italics)

The novel variants were prepared and purified, as shown above, and all enzymes could be purified. The variants were compared to the wt AroF. Data are shown in Supplementary Table S4, Figs. 3 and 4.

The two new variants at position E154 (S and Q) had similar specific activities as E154N and were also feedback-resistant toward Tyr. E154S, while showing less activity, was completely resistant toward 5 mM Tyr. The new variants at position P155 showed different behavior. P155M and P155T were not much different in sensitivity to Tyr as the wt enzyme but were clearly less active. P155V kept about 60% activity with 50 µM Tyr but was almost inactive at 5 mM. P155I was similar in its behavior to P155L. We also wanted to determine whether combinations of the feedback inhibition-resistant variants show even increased resistance toward Tyr. However, the combinations of E154/P 155 did not show additive resistance features (Supplementary Fig. S5).

## Discussion

We applied structure-based protein engineering to induce feedback resistance in AroF_cg_, the type I DAHPS from *C. glutamicum*, which is sensitive toward the presence of 50 μM Tyr (remaining activity < 40%) and becomes almost inactive in 5 mM Tyr. We initially predicted eight AroF_cg_ variants with single substitutions of inhibitor binding site residues, which were evaluated by two activity assays in vitro. Two of the variants yielded > 80% (E154N) and > 50% (P155L) remaining activity at 5 mM Tyr and showed > 50% specific activities compared to wt AroF_cg_ in the absence of Tyr. Evaluation of two and four further variants at positions 154 and 155, respectively, yielded E154S, which is completely resistant to 5 mM Tyr, and P155I, which behaves similarly to P155L.

Rational engineering for deregulation of feedback inhibition has often been pursued in the context of the shikimate pathway (Guo et al. 2019; Rajkumar and Morrissey 2020; Syukur Purwanto et al. 2018; Zhang et al. 2015) and other pathways (Chen et al. 2014; Yang et al. 2012). For this, residues involved in feedback inhibitor binding (Chen et al. 2014) or identified from evolutionary and physicochemical information (Yang et al. 2012) have been subjected to substitutions, or knowledge of feedback-resistant substitutions in homologous enzymes (Rajkumar and Morrissey 2020) has been exploited. Here, we pursued a consensus approach by drawing on structural modeling, sequence and structural comparisons, and knowledge of feedback-resistant variants in *E. coli* homologs. We computed folding free energy changes and predicted two types of putatively feedback-resistant variants: those where substitutions destabilize the inhibitor binding site (E154N, N156I, D163A, T220V, and D222A) and those where substitutions directly interfere with inhibitor binding (P155L, Q159A, S188F, and, again, D222A).

These variants were cloned, expressed, and evaluated with two enzyme activity assays that allow measuring the influence of low (50 µM) or high (5 mM) concentrations of Tyr, respectively.

The computed folding free energy changes suggested disfavorable variant stabilities for S188F (both methods, FoldX and Rosetta), D163A (FoldX), and Q159A (energy change barely negative for FoldX). These variant enzymes were subsequently found to be non-expressible (D163A), unstable (Q159A), or to display a greatly reduced specific activity (S188F). This points to the value of using such computations for identifying variants with stability issues, and FoldX seems to be more sensitive for this, in line with previous evaluations (Buss et al. 2018). Note, however, that multiple folding free energy predictors should be applied in parallel to reduce the likelihood of predicting false negatives, as would have happened for E154N when using FoldX alone. Even then, however, expressible and active variants could be excluded (E154Q, P155T). When performed on known feedback-resistant variants of AroG_ec_, a similar picture emerges (Supplementary Table S5).

Of the two types of predicted, putatively feedback-resistant variants, those where substitutions should destabilize the inhibitor binding site remained more sensitive at 50 μM Tyr (N156I, T220V, and D222A), although E154N, which also belongs to this class, kept more than 80% of its activity in the presence of 50 µM Tyr. The former cases may arise because of allosteric signal transmission by conformational changes from the inhibitor site to the active site, which has been identified previously for the Phe- and Tyr-inhibited DAHPS homologs from *E. coli* (Cui et al. 2019; Shumilin et al. 2002); such conformational changes may be facilitated if the inhibitor binding site becomes destabilized. By contrast, the E154-equivalent residue D146 in AroG_ec_ interacts with T149 upon Phe binding only (Shumilin et al. 2002), such that the substitution E154N may also impact Tyr binding in AroF_cg_, that way leading to the pronounced feedback inhibitor resistance of that variant. The closeness of the inhibitor binding and active sites, with a minimum distance of 8.5 Å, is likely also the reason why changes in the former impact the specific activity of E154N and P155L compared to wt AroF_cg_.

Finally, evaluation of two and four further variants at positions 154 and 155 confirmed the relevance of substituting E154 (see also Supplementary Fig. S6), as E154Q was only slightly inferior to E154N as to feedback resistance, whereas E154S was completely resistant. By contrast, only P155I, with a substitution the most similar to P155L, showed a marked feedback resistance, but changes in size (Val, Met) or polarity (Thr) abolished resistance.

In summary, evaluating eight plus six variants at eight positions of the inhibitor binding site of AroF_cg_ yielded two variants, each at positions 154 and 155, with at least ~ 50% to complete feedback inhibitor resistance at 5 mM Tyr. Our structure-based consensus approach including the evaluation of folding free energy changes, therefore, proved effective. A comparison of variants from AroF_cg_ and AroG_ec_ with mutations at structurally equivalent positions yields that their feedback resistance can differ (Supplementary Table S6), indicating that an enzyme-specific evaluation of variant predictions is required. Note that several of the newly detected feedback inhibition-resistant variants could not have been obtained by classical strain development as the necessary amino acid changes in *C. glutamicum* (e.g., Glu to Asn, Ser, or Leu) cannot be obtained by single-point mutations from a codon in position 154 (GAA for Glu) to either Asn (DNA codons either AAT or AAC), Ser (TCN or AGC/T), or Leu (CTN or TTA/G). Mutation of Pro at position 155 (codon CCA) to Leu (CTN or TTA/G) would be possible for one codon (CTA), but not to any of the Ile codons (AT C/A/T).

Possible applications of the newly found feedback-resistant variants could be the enhanced microbial production of shikimic acid, aromatic amino acids, or other products derived from the general aromatic pathway (Bongaerts et al. 2001; Ding et al. 2014; Ikeda 2006; Lee and Wendisch 2017; Martinez et al. 2015; Rodriguez et al. 2014; Sprenger 2007).

## Supplementary Information

Below is the link to the electronic supplementary material.

Supplementary file1 (PDF 477 KB)

## Data Availability

All data generated or analyzed during this study are included in this published article and its supplementary information file.
