# Supplementary material for: Protein engineering for feedback resistance in 3-deoxy-D-arabino-heptulosonate 7-phosphate synthase
Source: Appl Microbiol Biotechnol. 2022 Sep 16;106(19-20):6505–17. doi: 10.1007/s00253-022-12166-9 (PMC9529685; doi:10.1007/s00253-022-12166-9)
Supplement: Supplementary file 1 — Supplementary file1 (PDF 477 KB) [file 253_2022_12166_MOESM1_ESM.pdf]

Supplementary information for the article:

Protein engineering for feedback resistance in 3-deoxy-D-*arabino*-heptulosonate 7-phosphate synthase

Kumaresan Jayaraman[1#], Natalia Trachtmann[2#], Georg A. Sprenger[2§], Holger Gohlke[1,3]*

[1]Institute for Pharmaceutical and Medicinal Chemistry, Heinrich Heine University Düsseldorf, 40225 Düsseldorf, Germany

[2]Institute of Microbiology, University of Stuttgart, Allmandring 31, 70569 Stuttgart, Germany

[3]John von Neumann Institute for Computing (NIC), Jülich Supercomputing Centre (JSC), Institute of Biological Information Processing (IBI-7: Structural Biochemistry) & Institute of Bio- and Geosciences (IBG-4: Bioinformatics), Forschungszentrum Jülich GmbH, 52425 Jülich, Germany

# these researchers contributed equally to this manuscript

Supplemental Methods

Construction of a triple-negative strain of *Escherichia coli* K-12.

Wildtype strain *E. coli* K-12 LJ110 (Zeppenfeld et al. 2000) is prototrophic. Using a CRISPR-Cas method (Jiang et al. 2015), the chromosomal genes encoding the three DAHP synthase isozymes (AroF, AroG and AroH) were subsequently deleted, yielding strain NT1402 (Δ*aroF* Δ*aroG* Δ*aroH*; N Trachtmann and GA Sprenger, unpublished results). Strain NT1402 has a pleiotropic phenotype and is auxotrophic for the three aromatic amino acids (Phe, Trp, Tyr) as well as for shikimic acid and aromatic vitamins.

Expression of *aroF*$_{Cg}$ wild type and *aroF*$_{Cg}$ E154N mutant gene in *E. coli* NT1402.

Plasmid pJNTN-L (*lacI*q, P*tac*, kanamycin-resistance) is a cloning and expression vector essentially based on plasmid pJF119EH (Fürste et al. 1986) but has a kanamycin resistance instead of the ampicillin resistance (N Trachtmann and GA Sprenger, unpublished results). pJNTN-L vector was used to clone and express the *aroF*$_{Cg}$ wildtype and *aroF*$_{Cg}$ E154N variant, respectively. Strain NT1402 was transformed with the empty vector pJNTN-L, pJNTN-L-*aroF*$_{Cg}$, and pJNTN-L-*aroF*$_{Cg}$E154N, respectively. The selection was for resistance against kanamycin (50 mg/l) on LB agar plates.

Test for sensitivity against the antimetabolite, *m*-D,L-fluorotyrosine.

To test for sensitivity or resistance versus the structural analog of tyrosine and antimetabolite, *m*-fluoro-*DL*-tyrosine, freshly grown (LB medium) overnight cultures were centrifuged and washed in minimal medium (Tanaka et al. 1967). Then, about 10^8 cells each were spread out on minimal agar plates containing 5 g/l of glucose, kanamycin (50 mg/l), and 200 µM of IPTG to induce the expression of plasmid-borne *aroF* gene. Sterile paper filter discs were placed on top of the bacterial lawn cultures. Then, 10 µl of a 100 mM m-D,L-fluorotyrosine (Sigma-Aldrich) solution were pipetted onto the filter discs. Agar plates were incubated for 24 h at 37°C before the examination (see Fig S6).

Supplemental Tables

Table S1. Homology modeling of $AroF_{cg}$[a]

Template	Identity (%)	Similarity (%)	Coverage (%)
1KFL	52.5	94.3	92.6
3TQK	51.2	91.1	90.7
1OF6	49.3	92.1	95.4
6U8J	46.8	92.9	98.6
4UMA	52.4	92.5	90.2
5D04	50.1	92.4	93.7
5CKS	53.3	94.7	91.8
6AGM	47.3	91.1	90.7

[a] The template structures used to model $AroF_{cg}$ and the corresponding sequence identities, similarities, and coverages are listed.

Table S2. Primers used in this work

		Primers	Restriction site
Cloning of the *aro*F gene into pET28a vector	1	TTTT<u>*CATATG*</u>AGTTCTCCAGTCTCACTCGAAAAC	*Nde*I
	2	TTTT<u>*GGATCC*</u>TTACTTGGCTGCTGCTCG	*Bam*HI
Mutation E154N	3	CGAATTCCTCAATCCAAACAGCCCTCAGTACTACGCCGA C	
	4	GGCTGTTTGGATTGAGGAATTCGCAGCCGACTGGG	
Mutation D163A	5	AGTACTACGCCGCCACTGTCGCATGGGGAGCAATCG	
	6	CATGCGACAGTGGCGGCGTAGTACTGAGGGCTGTTTGG	
Mutation S188F	7	CTTCTGGGATGTTTATGCCAATTGGTTTCAAGAACGGAAC	
	8	ACCAATTGGCATAAACATCCCAGAAGCCAGCTGGCGGTGCAC	
Mutation D222A	9	CTTCGGAACCTCCGCCGACGGCGCGCTGAGCGTCGTGGAG	
	10	CGCGCCGTCGGCGGAGGTTCCGAAGAAGAAGTGTGGG	
Mutation P155L	11	GAATTCCTCGAATTGAACAGCCCTCAGTACTACGCCGAC	
	12	GAGGGCTGTTCAATTCGAGGAATTCGCAGCCGACTG	
Mutation N156I	13	TCCTCGAACCAATCAGCCCTCAGTACTACGCCGAC	
	14	TGAGGGCTGATTGGTTCGAGGAATTCGCAGCCG	
Mutation Q159A	15	CAAACAGCCCTGCATACTACGCCGACACTGTCGC	
	16	GCGAATTCCTCGAACCAAACAGCCCTGCATACTACGCC	
Mutation T220V	17	TCTTCTTCGGAGTTTCCGACGACGGCGCGCTGAGC	
	18	GTCGTCGGAAACTCCGAAGAAGAAGTGTGGGTTCTGG	
Mutation E154A	19	CGAATTCCTCGCACCAAACAGCCCTCAGTACTACGCCGAC	
	20	GGCTGTTTGGTGCGAGGAATTCGCAGCCGACTGGG	
Mutation E154R	21	CGAATTCCTCCGCCCAAACAGCCCTCAGTACTACGCCGAC	
	22	GGCTGTTTGGGCGGAGGAATTCGCAGCCGACTGGG	
Mutation E154D	23	CGAATTCCTCGACCCAAACAGCCCTCAGTACTACGCCGAC	
	24	GGCTGTTTGGGTCGAGGAATTCGCAGCCGACTGGG	
Mutation E154Q	25	CGAATTCCTCCAGCCAAACAGCCCTCAGTACTACGCCGAC	
	26	GGCTGTTTGGCTGGAGGAATTCGCAGCCGACTGGG	
Mutation E154C	27	CGAATTCCTCTGCCCAAACAGCCCTCAGTACTACGCCGAC	
	28	GGCTGTTTGGGCAGAGGAATTCGCAGCCGACTGGG	
Mutation E154G	29	CGAATTCCTCGGCCCAAACAGCCCTCAGTACTACGCCGAC	
	30	GGCTGTTTGGGCCGAGGAATTCGCAGCCGACTGGG	
Mutation E154H	31	CGAATTCCTCCACCCAAACAGCCCTCAGTACTACGCCGAC	
	32	GGCTGTTTGGGTGGAGGAATTCGCAGCCGACTGGG	
Mutation E154I	33	CGAATTCCTCATCCCAAACAGCCCTCAGTACTACGCCGAC	
	34	GGCTGTTTGGGATGAGGAATTCGCAGCCGACTGGG	
Mutation E154L	35	CGAATTCCTCCTGCCAAACAGCCCTCAGTACTACGCCGAC	
	36	GGCTGTTTGGCAGGAGGAATTCGCAGCCGACTGGG	
Mutation E154K	37	CGAATTCCTCAAGCCAAACAGCCCTCAGTACTACGCCGAC	
	38	GGCTGTTTGGCTTGAGGAATTCGCAGCCGACTGGG	
Mutation E154M	39	CGAATTCCTCATGCCAAACAGCCCTCAGTACTACGCCGAC	
	40	GGCTGTTTGGCATGAGGAATTCGCAGCCGACTGGG	
Mutation E154F	41	CGAATTCCTCTTCCCAAACAGCCCTCAGTACTACGCCGAC	
	42	GGCTGTTTGGGAAGAGGAATTCGCAGCCGACTGGG	
Mutation E154P	43	CGAATTCCTCCCACCAAACAGCCCTCAGTACTACGCCGAC	
	44	GGCTGTTTGGTGGGAGGAATTCGCAGCCGACTGGG	
Mutation E154S	45	CGAATTCCTCTCCCCAAACAGCCCTCAGTACTACGCCGAC	
	46	GGCTGTTTGGGGAGAGGAATTCGCAGCCGACTGGG	
Mutation E154T	47	CGAATTCCTCACCCCAAACAGCCCTCAGTACTACGCCGAC	
	48	GGCTGTTTGGGGTGAGGAATTCGCAGCCGACTGGG	
Mutation E154W	49	CGAATTCCTCTGGCCAAACAGCCCTCAGTACTACGCCGAC	
	50	GGCTGTTTGGCCAGAGGAATTCGCAGCCGACTGGG	

Mutation E154Y	51	CGAATTCCTCTACCCAAACAGCCCTCAGTACTACGCCGAC	
	52	GGCTGTTTGGGTAGAGGAATTCGCAGCCGACTGGG	
Mutation E154V	53	CGAATTCCTCGTGCCAAACAGCCCTCAGTACTACGCCGAC	
	54	GGCTGTTTGGCACGAGGAATTCGCAGCCGACTGGG	

Table S3. Genbank accession numbers for deposited nucleotide sequences of $AroF_{cg}$ variants

Variant	Accession number
aroF_E154N	ON263165
aroF_E154A	ON263166
aroF_E154R	ON263167
aroF_E154D	ON263168
aroF_E154C	ON263169
aroF_E154Q	ON263170
aroF_E154G	ON263171
aroF_E154H	ON263172
aroF_E154I	ON263173
aroF_E154L	ON263174
aroF_E154K	ON263175
aroF_E154M	ON263176
aroF_E154F	ON263177
aroF_E154P	ON263178
aroF_E154S	ON263179
aroF_E154T	ON263180
aroF_E154W	ON263181
aroF_E154Y	ON263182
aroF_E154V	ON263183
aroF_P155L	ON263184
aroF_P155T	ON263185
aroF_P155M	ON263186
aroF_P155I	ON263187
aroF_P155V	ON263188
aroF_N156I	ON263189
aroF_Q159A	ON263190
aroF_T220V	ON263191
aroF_D163A	ON263192
aroF_S188F	ON263193
aroF_D222A	ON263194

Table S4. DAHP Synthase activities in the presence or absence of effector Tyr

**Specific activity of the DAHP synthase (units/mg) with standard deviations**										
	aroF-wt	aroF-E154N	aroF-E154S	aroF-E154Q	aroF-P155L	aroF-P155M	aroF-P155T	aroF-P155I	aroF-P155V	aroF-Q159A
Continuous method										
w/o tyrosine	5.26±0.2	2.4± 0.1	1.76±0.3	2.42±0.3	3.16±0.1	3.84±0.05	6.17±0.4	3.96±0.1	5.35±0.1	4.10±0.5
50 µM tyrosine	2.10±0.2	2.00±0.1	1.82±0.4	2.03±0.1	2.63±0.5	1.15±0.18	0.94±0.01	3.10±0.01	3.30±0.3	3.22±0.1
Colorimetric method										
w/o tyrosine	4.97±0.3	2.5±0.07	1.56±0.1	3.72±0.1	4.56±0.2	1.22±0.1	2.54±0.1	3.96±0.3	4.51±0.3	1.79±0.1
5 mM tyrosine	0.07±0.04	2.06±0.1	1.74±0.08	2.36±0.08	2.37±0.13	0.029±0.01	0.02±0.01	1.94±0.14	0.35±0.1	0.38±0.0
**Residual enzyme activities of variants (in %) in the presence of different Tyr concentrations**										
	Remaining activity in % compared to the activity without tyrosine									
	aroF-wt	aroF-E154N	aroF-E154S	aroF-E154Q	aroF-P155L	aroF-P155M	aroF-P155T	aroF-P155I	aroF-P155V	aroF-Q159A
w/o tyrosine	100	100	100	100	100	100	100	100	100	100
50 µM tyrosine	39.9	83.6	102.9	83.7	83.3	30.1	15.2	78.3	61.7	78.4
5 mM tyrosine	1.3	82.5	111.4	63.3	52.0	2.4	0.8	49.1	7.7	21.4

Table S5: The changes in the folding free energy change (ΔΔ*G*) of known feedback-resistant variants of AroG$_{ec}$ at positions structurally equivalent to those investigated here in AroF$_{cg}$ [a]

**Variants**	**ΔΔ*G*[a]**	
	**FoldX**	**Rosetta**
D146N	0.54 ± 0.06	2.19 ± 0.26
M147I	0.39 ± 0.02	1.86 ± 0.25
Q151A	-0.26 ± 0.01	0.40 ± 0.23
S180F	0.29 ± 0.02	1.91 ± 0.53

[a] In kcal mol^{-1}. ΔΔ*G* (eq. 1) is predicted with FoldX and Rosetta. Given is the average ± SEM over *n* = 50 data points for FoldX and *n* = 10 for Rosetta. Variants in which at least one ΔΔ*G* value < 0 are considered stable (marked in italics).

Table S6: Comparison of variants from $AroF_{cg}$ and $AroG_{ec}$ with mutations at structurally equivalent positions with respect to feedback resistance

Variants		Biochemical properties
**$AroF_{cg}$**	**$AroG_{ec}$**	
E154N	D146N	Fbr[a] at 2 mM Phe in $AroG_{ec}$ (Kikuchi et al. 1997); fbr at 5 mM Tyr in $AroF_{cg}$.
P155L	M147I	Fbr at 2 mM Phe in $AroG_{ec}$ (Kikuchi et al. 1997); fbr at 5 mM Tyr in $AroF_{cg}$.
Q159A	Q151A	Fbr at 20 mM Phe in $AroG_{ec}$ (Yenyuvadee et al. 2021); strong fbr at 50 µM Tyr in $AroF_{cg}$, but moderate fbr at 5 mM Tyr.
S188F	S180F	Fbr at 20 mM Phe in $AroG_{ec}$ (Ger et al. 1994); fbr at 50 µM Tyr.

[a] Fbr: Feedback resistance

Supplemental Figures

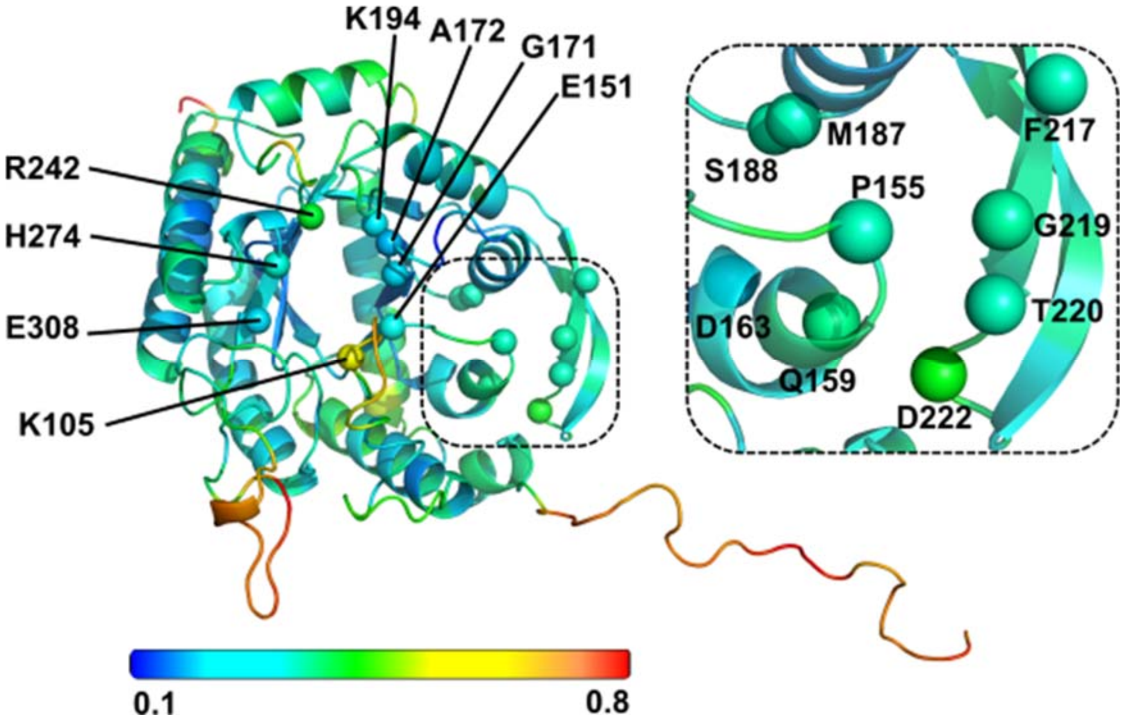

Figure S1. Structural model of AroF$_{cg}$. Structural model of AroF$_{cg}$ predicted with TopModel (Mulnaes et al. 2020) colored according to the residue-wise model quality assessment with TopScore (Mulnaes and Gohlke 2018). Blue colors indicate regions with high structural quality, red colors regions with low structural quality (see color scale). The catalytic and inhibitor site residues are depicted with spheres and labeled. The inhibitor binding region is shown with a blow-up in the right.

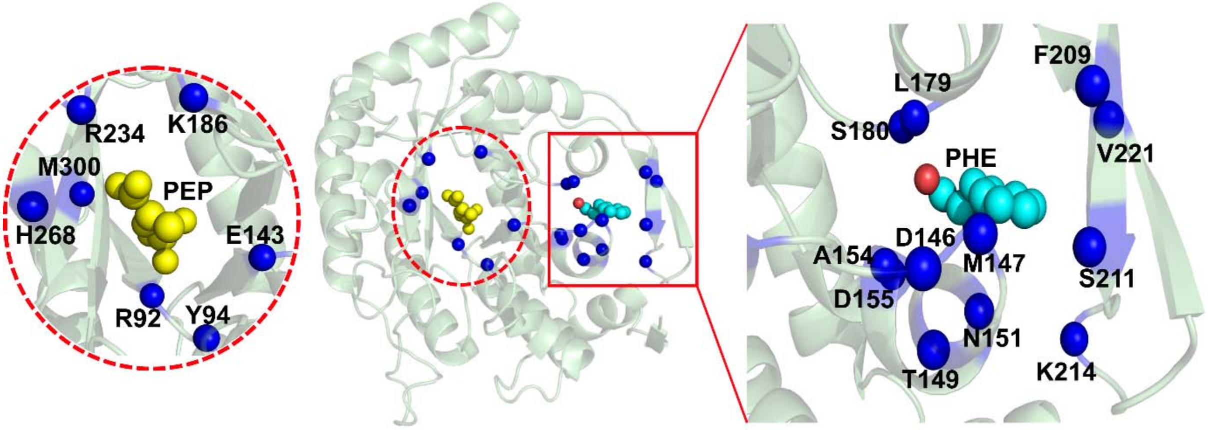

Figure S2. Structure of the Phe-sensitive class I DAHPS from *E. coli* (AroG$_{ec}$). The structure in the middle is AroG$_{ec}$ (PDB ID 1KFL), which was solved with PEP and Phe bound at the catalytic (circle) and regulatory (square) sites, respectively. A blow-up of the catalytic site is shown on the left, with C$_\alpha$ atoms of catalytic site residues depicted as blue spheres. The substrate PEP is illustrated with yellow spheres. A blow-up of the regulatory site is shown on the right, with C$_\alpha$ atoms of residues there shown with blue spheres. The bound Phe is represented with cyan spheres.

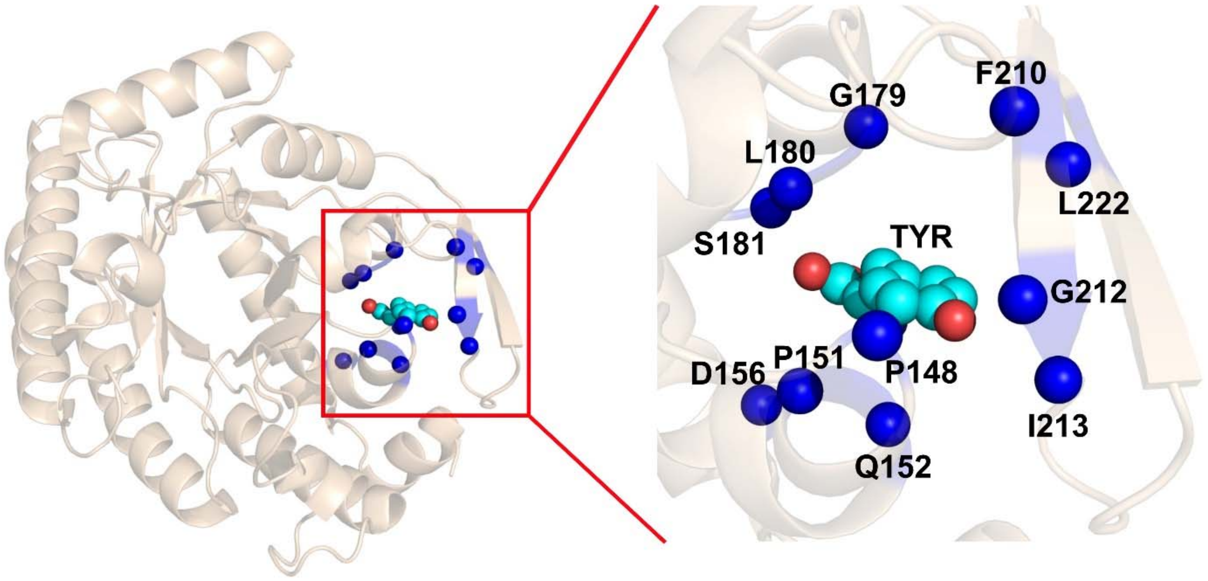

Figure S3. Structure of the Tyr-sensitive class I DAHPS from *E. coli* (AroF$_{ec}$). The structure of AroGec (PDB ID 6AGM) was resolved with Tyr bound at the inhibitor binding site (square). A blow-up of this site is shown on the right, with C$_\alpha$ atoms of residues of the site depicted as blue spheres. Tyr is illustrated in cyan spheres.

a) SDS-PAGE analysis

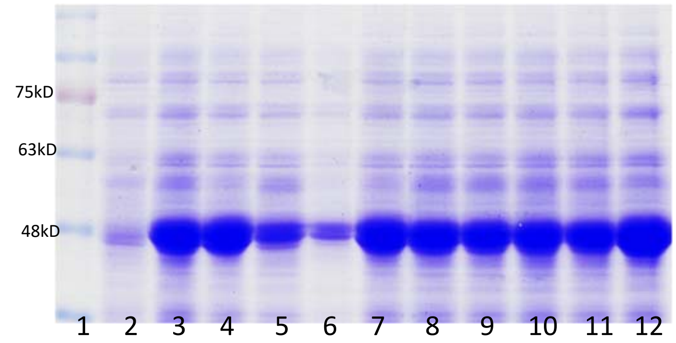

1. Protein Size Marker
2. Cell free extract BL21(DE3)pLys +pET28a
3. Cell free extract BL21(DE3)pLys +pET28a-aroF-wt
4. Cell free extract BL21(DE3)pLys +pET28a-aroF-E154N
5. Cell free extract BL21(DE3)pLys +pET28a-aroF-E154S
6. Cell free extract BL21(DE3)pLys +pET28a-aroF-E154Q
7. Cell free extract BL21(DE3)pLys +pET28a-aroF-P155L
8. Cell free extract BL21(DE3)pLys +pET28a-aroF-P155M
9. Cell free extract BL21(DE3)pLys +pET28a-aroF-P155T
10. Cell free extract BL21(DE3)pLys +pET28a-aroF-P155I
11. Cell free extract BL21(DE3)pLys +pET28a-aroF-P155V
12. Cell free extract BL21(DE3)pLys +pET28a-aroF-Q159A

b) Purification of AroF$_{Cg}$ enzyme variants by IMAC (Ni-NTA chromatography)

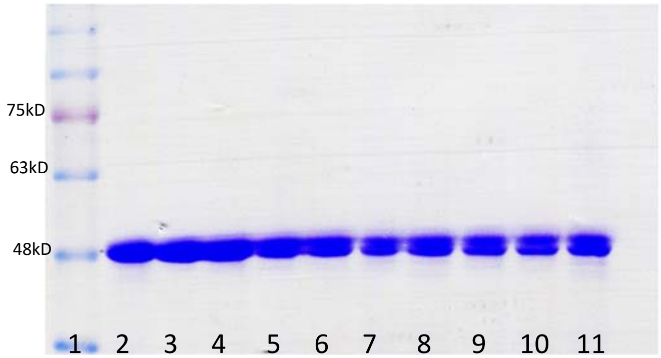

1. Marker
2. aroF-wt
3. aroF-E154N
4. aroF-E154S
5. aroF-E154Q
6. aroF-P155L
7. aroF-P155M
8. aroF-P155T
9. aroF-P155I
10. aroF-P155V
11. aroF-Q159A

Figure S4. Expression of AroF$_{Cg}$ wildtype and mutant genes in *E.coli* BL21(DE3)pLys-pET28a. Purification of protein variants N156I, S118F, T220V, and D222A was performed likewise (data not shown).

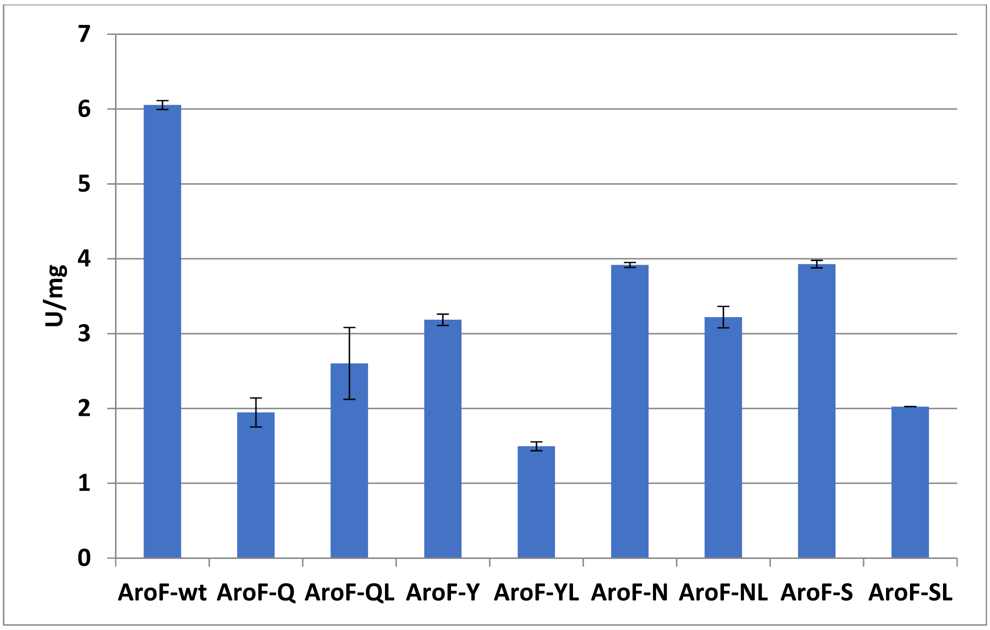

Figure S5. The specific activity of the purified proteins (single and double mutants) without tyrosine.

AroF-Q: AroF E154Q

AroF-QL: AroF E154Q P155L

AroF-Y: AroF E154Y

AroF-YL: AroF E154Y P155L

AroF-N: AroF E154N

AroF-NL: AroF E154N P155L

AroF-S: AroF E154S

AroF-SL: AroF E154S P155L

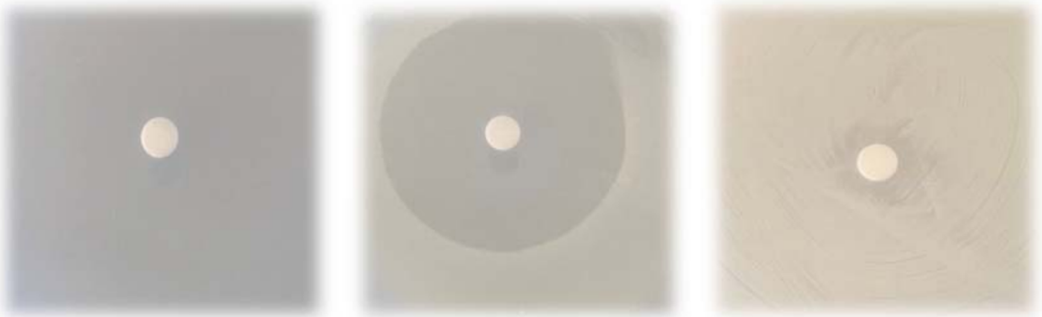

a) NT 1402 b) NT 1402 c) NT 1402

+ pJNTN-L + pJNTN-aroF_{Cg}-wt + pJNTN-aroF_{Cg}-E154N

Fig. S6 Effect of the antimetabolite, fluoro-tyrosine, on the growth of triple-negative *E. coli* K-12 mutant strain NT 1402 on MM agar plates. For details of strain and plasmid construction and preparation of cells for the assay, see Supplemental Methods. Growth on Tanaka minimal medium agar plates (0.5% glucose, 200 µM IPTG, 50 mg/l of kanamycin). 10 µl of a 100 mM *m*-fluoro-*DL*-tyrosine solution were pipetted onto the filter discs. Plates were incubated for 24 h at 37°C before examination. a) For the control strain NT1402 with the empty vector, no growth was observed on MM agar plates as the strain has a pleiotropic auxotrophy due to the triple mutation Δ*aroF* Δ*aroG* Δ*aroH*. b) and c) shows formation of bacterial lawn and different inhibition zones caused by the antimetabolite.

Supplemental References

Fürste JP, Pansegrau W, Frank R, Blöcker H, Scholz P, Bagdasarian M, Lanka E (1986) Molecular cloning of the plasmid RP4 primase region in a multi-host-range tacP expression vector. Gene 48: 119–131.

Ger YM, Chen SL, Chiang HJ, Shiuan D (1994) A single Ser-180 mutation desensitizes feedback inhibition of the phenylalanine-sensitive 3-deoxy-D-arabino-heptulosonate 7-phosphate (DAHP) synthetase in Escherichia coli. J Biochem 116(5):986-90. doi:10.1093/oxfordjournals.jbchem.a124657

Jiang Y, Chen B, Duan C, Sun B, Yang J, Yang S (2015) Multigene editing in the *Escherichia coli* genome via the CRISPR-Cas9 system. Appl Environm Microbiol 81:2506–2514

Kikuchi Y, Tsujimoto K, Kurahashi O (1997) Mutational analysis of the feedback sites of phenylalanine-sensitive 3-deoxy-D-arabino-heptulosonate-7-phosphate synthase of Escherichia coli. Appl Environ Microbiol 63(2):761-2 doi:10.1128/aem.63.2.761-762.1997

Mulnaes D, Gohlke H (2018) TopScore: Using Deep Neural Networks and Large Diverse Data Sets for Accurate Protein Model Quality Assessment. J Chem Theory Comput 14(11):6117-6126 doi:10.1021/acs.jctc.8b00690

Mulnaes D, Porta N, Clemens R, Apanasenko I, Reiners J, Gremer L, Neudecker P, Smits SHJ, Gohlke H (2020) TopModel: Template-Based Protein Structure Prediction at Low Sequence Identity Using Top-Down Consensus and Deep Neural Networks. J Chem Theory Comput 16(3):1953-1967 doi:10.1021/acs.jctc.9b00825

Tanaka S, Lerner SA, Lin ECC (1967) Replacement of a phosphoenolpyruvate-dependent phosphotransferase by a nicotinamide adenine dinucleotide-linked dehydrogenase for the utilization of mannitol. J Bacteriol 93:642–648

Yenyuvadee C, Kanoksinwuttipong N, Packdibamrung K (2021) Effect of Gln151 on L-phenylalanine feedback resistance of AroG isoform of DAHP synthase in Escherichia coli. ScienceAsia 47:40-46 doi:10.2306/scienceasia1513-1874.2021.004

Zeppenfeld T, Larisch C, Lengeler JW, Jahreis K (2000) Glucose transporter mutants of *Escherichia coli* K-12 with changes in substrate recognition of IICBGlc and induction behavior of the *ptsG* gene. J Bacteriol 182:4443–4452
